# Blood Aluminum Levels in Patients with Hemodialysis and Peritoneal Dialysis

**DOI:** 10.3390/ijerph19073885

**Published:** 2022-03-24

**Authors:** Po-Hsun Chuang, Kai-Fan Tsai, I-Kuan Wang, Ya-Ching Huang, Lan-Mei Huang, Shou-Hsuan Liu, Cheng-Hao Weng, Wen-Hung Huang, Ching-Wei Hsu, Wen-Chin Lee, Tzung-Hai Yen

**Affiliations:** 1 Department of Nephrology, Clinical Poison Center, Chang Gung Memorial Hospital, Linkou 333, Taiwan; tony0430@cgmh.org.tw (P.-H.C.); mei1191@cgmh.org.tw (L.-M.H.); mcpom@hotmail.com (S.-H.L.); drweng@seed.net.tw (C.-H.W.); williammedia@yahoo.com.tw (W.-H.H.); wei2838@gmail.com (C.-W.H.); 2 College of Medicine, Chang Gung University, Taoyuan 333, Taiwan; b9302095@cgmh.org.tw (K.-F.T.); hycymm@cgmh.org.tw (Y.-C.H.); leewenchin@gmail.com (W.-C.L.); 3 Division of Nephrology, Department of Internal Medicine, Kaohsiung Chang Gung Memorial Hospital, Kaohsiung 833, Taiwan; 4 Department of Nephrology, China Medical University Hospital, Taichung 404, Taiwan; ikwang@mail.cmuh.org.tw; 5 College of Medicine, China Medical University, Taichung 406, Taiwan; 6 Department of Laboratory Medicine, Chang Gung Memorial Hospital, Linkou 333, Taiwan

**Keywords:** blood aluminum levels, hemodialysis, peritoneal dialysis, aluminum toxicity

## Abstract

Background. This retrospective observational study attempted to examine the prevalence of abnormal blood aluminum levels in dialysis patients, and to explore the association of pathogenic factors, such as demographic, clinical, laboratory as well as the use of phosphate binding drugs, drugs for secondary hyperparathyroidism and erythropoiesis-stimulating drugs with the blood aluminum levels. Methods. The study included 1175 patients (874 hemodialysis and 301 peritoneal dialysis), recruited from Chang Gung Memorial Hospital in November 2020. Patients were stratified into two groups by their blood aluminum levels, as normal (<2 µg/dL, *n* = 1150) or abnormal (≥2 µg/dL, *n* = 25). Results. The patients aged 60.4 ± 13.2 years and were dialyzed for 8.6 ± 8.1 years. The average blood aluminum level was 1.0 ± 0.4 µg/dL. Patients with abnormal blood aluminum levels received more sevelamer than patients with normal blood aluminum level (*p* = 0.014). Patients with abnormal blood aluminum levels had higher platelet count (*p* = 0.001), triglyceride (*p* < 0.001) and total iron binding capacity (*p* = 0.003) than patients with normal blood aluminum levels. Moreover, the cardiothoracic ratio was higher in patients with abnormal blood aluminum levels than patients with normal blood aluminum levels (*p* = 0.003). Conclusions. The prevalence of abnormal blood aluminum levels was low at 2.2%. Nevertheless, the linking of cardiothoracic ratio of more than 0.5 as well as elevated blood platelet count and triglyceride level with blood aluminum levels are interesting, and warranted more researches in this area.

## 1. Introduction

In the general population, exposure to aluminum usually happens through the foodstuffs (processed foods), drinking water, and aluminum-containing medicines, aluminum cookware or cosmetic products (for example antiperspirants, sun creams and toothpaste) [1]. The intake of aluminum from food and water is relatively little in comparison with aluminum-containing medicines. Inhalation and ingestion (via food and water) are the two principal routes through which aluminum enters into human body, but aluminum can also be absorbed through the skin [2,3]. However, the penetration rate of aluminum following dermal product of antiperspirants is exceedingly low [2]. For aluminum industry workers, there is another possibility for inhalation contact in the course of refining of primary metal and in secondary industries that fabricate aluminum goods (for example aircraft, automotive, and metal goods) and aluminum welding [4]. Tobacco smoke may also add to the concentration of aluminum in the air [5].

Around 1.5 to 2% of inhaled and 0.01 to 5% of ingested aluminum is absorbed, and the absorbed aluminum is excreted largely in the urine [4]. For the general population, aluminum is not bioaccumulated to a great extent [5]. Nevertheless, dialysis population should be considered as an exception. Accumulation of aluminum in dialysis population might arise from environmental or dietary exposure or contaminated dialysate. Chronic dialysis patients are at great odds for excess of aluminum, because they are subjected to high volumes of dialysate, and therefore even otherwise trivial concentration gradients of aluminum between blood and dialysate may produce substantial toxicity [6]. Moreover, absence of native kidney clearance in dialysis patients may also be prone to excess aluminum even not present in dialysate [6]. Patients undertaking hemodialysis thrice per week are exposed to 300 to 600 L of water based on the dialysis prescription [7], and patients undergoing peritoneal dialysis also have large volume of dialysate exposure depending on their residual kidney function, solute clearance and peritoneal dialysis modalities. Therefore, aluminum accumulation is a important concern in dialysis population because aluminum poisoning triggers many medical complications such as osteomalacia, low levels of parathyroid hormone, anemia, increased erythropoietin needs [8,9], dialysis encephalopathy [10,11,12,13] and increased mortality [14,15]. Nowadays, with the elimination of aluminum from water used for dialysis by water purification techniques and use of nonaluminum-containing phosphate binding agents, aluminum toxicity in dialysis patients is comparatively infrequent [16,17]. In a recent study of 14,919 American dialysis patients, Schifman and Luevano [18] reported a decreasing trend of blood aluminum levels in their cohort patients, and the percentage of abnormal blood aluminum levels (>2 µg/dL) also dropped from 31.5% in 2000 to 2.0% in 2015.

Given the importance of aluminum toxicity in dialysis population, this study aimed to examine the prevalence of abnormal blood aluminum levels in hemodialysis and peritoneal patients. Besides, our study attempted to analyze the association of potential pathogenic factors, such as baseline demographic, clinical, laboratory as well as the use of phosphate binding drugs, drugs for secondary hyperparathyroidism and erythropoiesis-stimulating drugs with the blood aluminum levels.

## 2. Materials and Methods

### 2.1. Patients

A total of 1175 dialysis patients (874 hemodialysis and 301 peritoneal dialysis) were recruited from the dialysis unit of Chang Gung Memorial Hospital in November 2020. Only patients who received hemodialysis or peritoneal dialysis for more than 6 months, aged 18 years and above, and had blood tests of aluminum level were included to the study. Patients with neoplasms, infections, or who were admitted or underwent operation in the prior 3 months were removed from analysis.

### 2.2. Groups

Patients were categorized into two groups by their blood aluminum levels as normal (<2 µg/dL, *n* = 1150) or abnormal (≥2 µg/dL, *n* = 25). This cutoff value was chosen according to the Clinical Practice Guidelines of National kidney Foundation [19].

### 2.3. Dialysis Prescription

Hemodialysis was accomplished with single-use hollow-fiber artificial kidneys fitted with modified cellulose, polyamide, or polysulfone membranes [20]. The dialysate was a standard ionic composition with bicarbonate-based buffer. A reverse osmosis technique was used for water purification. Peritoneal dialysis prescriptions were depended on peritoneal equilibration tests of peritoneal membrane characteristics. Intermittent therapy was arranged to patients with high membrane transport, and continuous therapy, to those with average or low membrane transport [21]. Low-calcium, icodextrin-based or standard dialysates containing glucose were prescribed according to the patients’ peritoneal transport characteristics and blood calcium levels to maintain adequate ultrafiltration and normal calcium levels.

### 2.4. Measurement of Blood Aluminum Levels

To ensure that the patients were not exposed to aluminum-contaminated water and dialysate during hemodialysis, two samples of dialysate were obtained from the inlets and outlets of the dialysate part of the dialyzers into aluminum-free plastic tubes [22]. Blood samples were collected and centrifuged to separate serum. All samples were deproteinized using trichloroacetic acid and microwave irradiation before measurements. All steps of sample preparation were conducted under a laminar flow hood. Aluminum was quantified by graphite furnace atomic absorption spectrometry with Zeeman background correction on a PerkinElmer 5100 (Norwalk, CT, USA). Distilled and de-ionized water was applied throughout the procedures. An aluminum standard solution containing 1000 mg/L (Merck, Germany) was used to prepare the working standard solutions. Nitric acid (HNO_3_, Merck, Germany) was purified by sub-boiling distillation. All laboratory ware (pipette tips, volumetric flasks, etc.) were immersed for at least 48 h in a 10% (*v/v*) HNO_3_/ethanol solution and washed with purified water shortly before use. This study used both internal and external quality-control procedures, and the results were reliably satisfactory. A certified commercially prepared product (Seronorm Trace Elements, Sero AS, Billingstads, Norway) was used to examine intra-batch accuracy and to ensure inter-batch standardization. The intra- and inter-batch coefficients of variation for the aluminum measurements were ≤5.0%. The detection limit was 0.1 μg/L. External quality control was maintained via participation in the National Quality Control Program conducted by the local health authority.

### 2.5. Statistical Analysis

All the data were tested for normality of distribution and equality of standard deviations before analysis. The quantile–quantile plot and Kolmogorov–Smirnov test were used to check the normality of distribution. The Levene test was used to check the equality of variance. Student’s *t*-test was used to analyze the quantitative parameters, and Chi-square or Fisher’s exact test, for categorical parameters. A *p* value of less than 0.05 was statistically significant. Data were evaluated with IBM SPSS Statistics Version 20.0.

## 3. Results

The patients aged 60.4 ± 13.2 years and were dialyzed for 8.6 ± 8.1 years (Table 1). None of the patients were aluminum industry worker. Systemic disease such hypertension (81.5%), diabetes mellitus (38.6%) and cardiovascular disease (16.1%) were prevalent in these patients. Furthermore, chronic glomerulonephritis (44.4%) diabetes mellitus (33.7%) and hypertension (7.8%) accounted for the leading causes of kidney failure in these patients. None of the patients with abnormal blood aluminum levels took aluminum hydroxide for phosphate control. Patients with abnormal blood aluminum levels received more sevelamer than patients with normal blood aluminum levels (20.0% vs. 5.7%, *p* = 0.014). Otherwise, there were no significant differences in baseline demographic data between two groups.

As shown in Table 2, the average blood aluminum level was 1.0 ± 0.4 µg/dL. Blood tests revealed that patients with abnormal blood aluminum levels had higher platelet count (250.7 ± 107.6 vs. 202.9 ± 71.7 10^3^/µL, *p* = 0.001), triglyceride (255.6 ± 180.9 vs. 167.8 ± 107.4 mg/dL, *p* < 0.001) and total iron binding capacity (288.8 ± 55.0 vs. 257.0 ± 52.7 µg/dL, *p* = 0.003) than patients with normal blood aluminum levels. Moreover, the cardiothoracic ratio was higher in patients with abnormal blood aluminum levels than patients with normal blood aluminum levels (0.5 ± 0.8 vs. 0.5 ± 0.8, *p* = 0.003). Otherwise, there were no significant differences in laboratory data between two groups.

## 4. Discussion

The average blood aluminum level of the dialysis patients was 1.0 ± 0.4 µg/dL, below the cutoff value set by National Kidney Foundation. As shown in Table 3 [14,17,18,22,23,24,25,26,27,28,29,30,31,32,33,34,35,36], there is a decreasing trend of blood aluminum levels in the dialysis units throughout the world. In the earlier studies, Neiva et al. [36] presented that the mean blood aluminum levels of Brazil hemodialysis patients was 4.5 ± 2.9 µg/dL, and Kazi et al. [33] presented that blood aluminum levels of 22.0 ± 2.5 µg/dL in Pakistan hemodialysis patients. Yue et al. [30] reported a mean blood aluminum level of 4.8 ± 2.0 µg/dL in Canada peritoneal dialysis patients. Compared with Hsu et al. [22] study from Chang Gung Memorial Hospital, the percentage of patients with abnormal blood aluminum levels dropped from 13.7% in 2018 to 2.2% in 2021. It might be due to the elimination of aluminum from water used for dialysis by reverse osmosis technique and use of nonaluminum-containing phosphate binders [17]. Table 3 also shows a large variations in the cutoff value of normal blood aluminum levels among different groups [37]. Importantly, the National Kidney Foundation Kidney Disease Outcomes Quality Initiative (KDOQI) Clinical Practice Guidelines recommended that physicians should take actions to decrease the blood aluminum levels in patients on chronic hemodialysis when it surpasses 2 µg/dL [19]. In addition, the Kidney Disease: Improving Global Outcomes (KDIGO) Clinical Practice Guidelines advised against chronic use of aluminum-containing binders to avoid aluminum accumulation [38].

The prevalence rate of abnormal blood aluminum levels was low (2.2%). None of the patients with abnormal blood aluminum levels took aluminum hydroxide for phosphate control. Furthermore, reverse osmosis technique is applied for water purification in our dialysis unit. Therefore, this raises the question of what sources of the aluminum exposure are. There is no exact explanation for the abnormal blood aluminum levels in our dialysis unit. However, the abnormal blood aluminum levels could possibly be explained by consumption of aluminum-contaminated medicines, consumption of aluminum-containing foods or aluminum contamination of food during culinary preparation. First, according to Bohrer et al. [39], aluminum exists as an adulteration in many raw materials used to produce medicines habitually used by dialysis patients. Unfortunately, the raw materials have warranty bulletins confirming their purity grade, but no limits for aluminum in these specifications. The medicines included calcium carbonate, vitamin B complex, folic acid, calcitriol, erythropoietin, iron sucrose, furosemide, heparin, etc., [39]. Second, excessive intake of processed foods with aluminum-containing food additives is considered. Aluminum-containing food additives are permitted to be used as a leavening agent in steamed cake/bread and bakery goods, anticaking agent in powder mix, coloring agent in confections, and as a firming agent in jellyfish. The European Food Safety Authority has derived a tolerable weekly intake of 1.0 mg/kg body weight for aluminum [40]. In a study, You et al. [41] demonstrated that the mean weekly dietary aluminum exposure in Taiwanese people was 1.071 mg/kg body weight. As the volume of aluminum-containing food intake in the Taiwanese total diet is rising, the researchers discovered that cake/waffle, kelp, snacks, and bread items were the main providers to aluminum exposure in Taiwanese people. In another European Total-Diet-Study Exposure project, Tietz et al. [42] revealed that the mean weekly aluminum exposure resulting from food intake was around 50% of the tolerable weekly intake of 1 mg/kg body weight set by European Food Safety Authority. Third, aluminum contamination of food during culinary preparation is considered. Whereas most researches proposed that cooking in aluminum cookware is safe, others warn that it might cause abnormal blood aluminum levels, especially in patients with chronic kidney disease [43]. Notably, to avoid aluminum contamination of food from aluminum foil, the Taiwan Food and Drug Administration [44] has advised public not to let acidic sauces contact the aluminum foil during barbecuing. Instead, it is advised to add the acidic sauces after the ingredients are cooked.

The cardiothoracic ratio was higher in patients with abnormal blood aluminum levels than patients with normal blood aluminum levels (*p* = 0.003). Higher cardiothoracic ratio was associated with higher risk for all-cause mortality in patients undergoing hemodialysis [45] and peritoneal dialysis [46]. Ghorbel et al. [47] presented that aluminum treatment created significant cardiotoxicity with oxidative damage in experimental animals. In another animal study, Novaes et al. [48] proved that heart was vulnerable to aluminum toxicity. Long-term aluminum treatment produced genomic DNA oxidation, structural irregularities of myocardium, causing widespread parenchymal loss, stromal enlargement, inflammatory cell infiltrations, collagen deposition, breakdown of collagen network, decreased myocardial vascularization, mitochondrial swelling, sarcomere disorder, myofilament separation, and fragmentation of cardiomyocytes. At the end, the cardiac stroma exhibited a compensatory enlargement, triggering constant pathological cardiac remodeling. In a study of 547 hemodialysis patients, Wang et al. [24] also identified a significant association between blood aluminum levels and cardiomegaly (cardiothoracic ratio more than 0.5).

Besides cardiothoracic ratio, there are some findings in this study but needed more researches to carify the significances. The association between blood aluminum levels and sevelamer usage was uncertain. This medicine is not a direct source of aluminum. Nevertheless, dialysis patients who suffered high blood aluminum levels might prone to use sevelamer for phosphate control. No medical studies demonstrated an association between sevelamer usage and blood aluminum levels. Moreover, there is no clear explanation for the association between blood aluminum levels and platelet count, although an increase of platelet count was observed in aluminum chloride-treated animals [49]. Similarly, no clear explanation for the association between blood aluminum and triglyceride level, although an increase of blood triglyceride level was observed in aluminum chloride-treated rats [50,51].

For the general population, oral exposure to aluminum is usually not harmful. On the other hand, dialysis population is susceptible to the aluminum accumulation and toxic effects of aluminum. Our previous study [25] proved that blood aluminum levels, even when in an seemingly suitable level (<2 µg/dL), was correlated with augmented mortality in chronic hemodialysis patients. The prevalence rate of high blood aluminum levels in this study was low (2.2%). Nevertheless, the linking of cardiothoracic ratio of more than 0.5 as well as elevated blood platelet count and triglyceride level with blood aluminum levels are stimulating and necessitated more studies in this field. Limitations of the current study comprised small sample size and lacking aluminum-containing medicine and diet evaluation.

## 5. Conclusions

The average blood aluminum levels of chronic dialysis patients were 1.0 ± 0.4 µg/dL. Nevertheless, the prevalence of abnormal blood aluminum levels was low at 2.2%.

## Figures and Tables

**Table 1:** Baseline demographics of hemodialysis and peritoneal dialysis patients, stratified according to their blood aluminum levels (*n* = 1175).

Variable	All Patients (*n* = 1175)	Normal Blood Aluminum Group (*n* = 1150)	Abnormal Blood Aluminum Group (*n* = 25)	*p* Value
Baseline				
Age (year)	60.4 ± 13.2	60.5 ± 13.2	56.7 ± 14.5	0.155
Male, *n* (%)	597 (50.8)	583 (50.7)	14 (56.0)	0.688
Smoking habit, *n* (%)	213 (18.2)	210 (18.4)	3 (12.0)	0.601
Body mass index (kg/m^2^)	23.9 ± 4.3	23.8 ± 4.2	25.0 ± 5.5	0.191
Duration of dialysis (year)	8.6 ± 8.1	8.6 ± 8.1	10.8 ± 7.5	0.176
				
Dialysis modality				0.149
Hemodialysis, *n* (%)	874 (74.4)	860 (74.7)	14 (60.9)	
Peritoneal dialysis, *n* (%)	301 (25.6)	292 (25.3)	9 (39.1)	
				
Systemic disease				
Hypertension, *n* (%)	954 (81.5)	937 (81.8)	17 (68.0)	0.112
Diabetes mellitus, *n* (%)	452 (38.6)	443 (38.7)	9 (36.0)	0.839
Cardiovascular diseases, *n* (%)	188 (16.1)	184 (16.1)	4 (16.0)	1.000
				
Etiology of kidney failure				0.647
Chronic glomerulonephritis, *n* (%)	522 (44.4)	508 (44.2)	14 (56.0)	
Diabetes mellitus, *n* (%)	396 (33.7)	392 (34.1)	4 (16.0)	
Hypertension, *n* (%)	92 (7.8)	89 (7.7)	3 (12.0)	
Polycystic kidney diseases, *n* (%)	28 (2.4)	27 (2.3)	1 (4.0)	
Drug, *n* (%)	9 (0.8)	9 (0.8)	0 (0)	
Obstructive uropathy, *n* (%)	41 (3.5)	40 (7.4)	1 (4.0)	
Others, *n* (%)	87 (7.4)	85 (7.4)	2 (8.0)	
				
Phosphate binding drugs				
Calcium carbonate, *n* (%)	727 (61.9)	714 (62.1)	13 (52.0)	0.306
Calcium acetate, *n* (%)	289 (24.6)	281 (24.4)	8 (32.0)	0.357
Aluminum hydroxide, *n* (%)	2 (0.2)	2 (0.2)	0 (0)	1.000
Sevelamer, *n* (%)	71 (6.0)	66 (5.7)	5 (20.0)	0.014 *
Lanthanum, *n* (%)	85 (7.2)	84 (7.3)	1 (4.0)	1.000
				
Drugs for secondary hyperparathyroidism				
Calcitriol, *n* (%)	616 (52.4)	601 (52.3)	15 (60.0)	0.545
Cinacalcet, *n* (%)	56 (4.8)	55 (4.8)	1 (4.0)	1.000
				
Erythropoiesis-stimulating drugs				
Erythropoietin dose, unit/month	19,845.9 ± 5375.7	19,853.0 ± 5384.0	19,520.0 ± 5075.4	0.759

Note: Patients were stratified into two groups by their blood aluminum levels, as normal blood aluminum group (<2 µg/dL) or abnormal blood aluminum group (≥2 µg/dL). This cutoff value was chosen according to the Clinical Practice Guidelines of National kidney Foundation [19]. Continuous variables were expressed as means and standard deviations, while categorical variables were expressed as numbers and percentages in parenthesis. * *p* < 0.05.

**Table 2:** Laboratory findings of hemodialysis and peritoneal dialysis patients, stratified according to their blood aluminum levels (*n* = 1175).

Variable	All Patients (*n* = 1175)	Abnormal Blood Aluminum Group (*n* = 25)	Normal Blood Aluminum Group (*n* = 1150)	*p* Value
Hemogram				
White blood cell count (10^3^/µL)	6.7 ± 2.2	7.1 ± 2.5	6.6 ± 2.2	0.321
Red blood cell count (×10^6^/µL)	3.4 ± 0.6	3.5 ± 0.8	3.4 ± 0.6	0.493
Hemoglobin (g/dL)	10.4 ± 1.3	10.6 ± 2.1	10.3 ± 1.3	0.422
Hematocrit (%)	31.1 ± 4.1	32.1 ± 6.2	31.1 ± 4.1	0.238
Mean corpuscular volume (fL)	91.9 ± 7.9	90.0 ± 8.3	91.9 ± 7.9	0.240
Platelet (×10^3^/µL)	203.9 ± 72.9	250.7 ± 107.6	202.9 ± 71.7	0.001 **
				
Biochemistry				
Aluminum (µg/dL)	1.0 ± 0.4	2.8 ± 1.2	1.0 ± 0.3	<0.001 ***
Albumin (mg/dL)	3.9 ± 0.4	4.0 ± 0.4	3.9 ± 0.4	0.460
Aspartate aminotransferase (U/L)	22.5 ± 14.3	23.0 ± 7.7	22.5 ± 14.4	0.847
Alanine aminotransferase (U/L)	20.4 ± 14.2	22.1 ± 8.5	20.3 ± 14.3	0.535
Alkaline phosphatase (U/L)	83.4 ± 65.8	101.7 ± 27.8	83.0 ± 64.4	0.155
Cholesterol (mg/dL)	164.0 ± 40.5	161.7 ± 28.5	164.1 ± 40.8	0.769
Triglyceride (mg/dL)	169.6 ± 110.1	255.6 ± 180.9	167.8 ± 107.4	<0.001 ***
Fasting glucose (mg/dL)	138.9 ± 66.1	131.8 ± 44.8	139.0 ± 66.5	0.590
Blood urea nitrogen (mg/dL)	70.3 ± 18.7	65.5 ± 19.9	70.4 ± 18.7	0.197
Creatinine (mg/dL)	10.6 ± 2.7	10.1 ± 2.9	10.6 ± 2.7	0.366
Uric acid (mg/dL)	6.6 ± 1.4	6.3 ± 1.5	6.6 ± 1.4	0.419
Sodium (meq/L)	138.3 ± 3.1	137.5 ± 3.8	138.3 ± 3.1	0.226
Potassium (meq/L)	4.7 ± 0.8	4.6 ± 1.0	4.7 ± 0.8	0.534
Calcium (mg/dL)	9.2 ± 0.9	9.5 ± 0.7	9.2 ± 0.9	0.066
Phosphorus (mg/dL)	5.2 ± 1.3	5.2 ± 1.1	5.2 ± 1.3	0.889
Iron (µg/dL)	70.0 ± 29.8	70.6 ± 30.9	70.0 ± 29.8	0.924
Total iron binding capacity (µg/dL)	257.7 ± 52.9	288.8 ± 55.0	257.0 ± 52.7	0.003 **
Transferrin saturation (%)	27.9 ± 12.3	24.6 ± 10.3	27.9 ± 12.3	0.173
Ferritin (ng/mL)	488.1 ± 472.7	463. 1 ± 392.5	488.7 ± 474.5	0.790
Intact parathyroid hormone (pg/mL)	307.8 ± 355.4	363.6 ± 347.1	306.6 ± 355.6	0.428
Normalized protein catabolic rate (g/kg/day)	1.2 ± 0.4	1.1 ± 0.3	1.2 ± 0.1	0.263
				
Chest radiography				
Cardiothoracic ratio	0.5 ± 0.1	0.5 ± 0.8	0.5 ± 0.1	0.003 **

Note: Patients were stratified into two groups by their blood aluminum levels, as normal blood aluminum group (<2 µg/dL) or abnormal blood aluminum group (≥2 µg/dL). This cutoff value was chosen according to the Clinical Practice Guidelines of National kidney Foundation [19]. Continuous variables were expressed as means and standard deviations, while categorical variables were expressed as numbers and percentages in parenthesis. ** *p* < 0.01, *** *p* < 0.001.

**Table 3:** Published studies of blood aluminum levels in chronic dialysis patients.

Study	Year	Area	Sample Size	Dialysis Modality	Blood Aluminum Levels, µg/dL	Cutoff Value of Normal Blood Aluminum Levels, µg/dL	Patient with Abnormal Blood Aluminum Levels, %
Present study	2022	Taiwan	1175	Hemodialysis 874 (74.4%); peritoneal dialysis 301 (25.6%)	1.0 ± 0.4	2.0	2.2
Humudat et al. [23]	2020	Iraq	320	Hemodialysis	21.0 ± 16.0	2.0	81.0
Hsu et al. [22]	2018	Taiwan	866	Hemodialysis	0.9	2.0	13.7
Tsai et al. [14]	2018	Taiwan	636	Hemodialysis	0.6	0.6	49.4
Schifman et al. [18]	2018	USA	14 919	Hemodialysis and peritoneal dialysis	0.8 (2015)	2.0	31.5 (2000); 2.0 (2015)
Wang et al. [24]	2017	Taiwan	547	Hemodialysis	0.7	0.6	Not mentioned
Hsu et al. [25]	2016	Taiwan	954	Hemodialysis	1.0 ± 0.7	2.0	Not mentioned
Sharma et al. [26]	2015	Australia	755	Hemodialysis 582 (77.0%); peritoneal dialysis 119 (15.8%)	1.1 ± 0.8	2.0	8.1
Cheng et al. [27]	2014	Taiwan	176	Hemodialysis 161 (92.0%); peritoneal dialysis 15 (8.0%)	1.3 ± 0.6	2.0	21.6
Edalat-Nejad et al. [28]	2014	Iran	136	Hemodialysis	1.6 ± 1.4	2.0	16.2
Christie et al. [29]	2011	Canada	111	Hemodialysis	0.6	2.0	Not mentioned
Yue et al. [30]	2011	Canada	83	Peritoneal dialysis	4.8	5.4	Not mentioned
Sandhu et al. [31]	2011	USA	589	Hemodialysis	1.0	Not mentioned	4.2
Cárdenas et al. [32]	2010	Colombia	63	Hemodialysis	2.65	Not mentioned	Not mentioned
Kazi et al. [33]	2008	Pakistan	100	Hemodialysis	22.0 ± 2.5	Not mentioned	Not mentioned
Chu et al. [34]	2007	USA	40	Hemodialysis	1.1	Not mentioned	Not mentioned
Gault et al. [17]	2005	UK	1626	Hemodialysis	1.3	6.0	1.8
Skarupskiene et al. [35]	2003	Lithuania	265	Hemodialysis	2.7 ± 4.4	3.0	24.9
Neiva et al. [36]	2002	Brazil	55	Hemodialysis	4.5 ± 2.9	Not mentioned	Not mentioned

## Data Availability

The datasets used and analyzed in this study are available from the corresponding author upon request.
